# Is condyle morphology a factor for anterior temporomandibular disc displacement?

**DOI:** 10.55730/1300-0144.5501

**Published:** 2022-06-18

**Authors:** İlkay ÇAMLIDAĞ, Aslı TANRIVERMİŞ SAYIT, Muzaffer ELMALI

**Affiliations:** Department of Radiology, Faculty of Medicine, Ondokuz Mayıs University, Samsun, Turkey

**Keywords:** Temporomandibular joint disorders, magnetic resonance imaging, mandibular condyle, morphology, disc displacement

## Abstract

**Background/aim:**

To investigate morphological features of the mandibular condyle and its association with anterior temporomandibular disc displacement on sagittal oblique MRI plane.

**Materials and methods:**

One hundred and twenty patients with temporomandibular MRI examination were retrospectively involved in the study. Patients aged less than 18 years and those with severe osteoarthritis, posterior disk displacement, tumor, abscess, history of a rheumatic disease, facial trauma, and motion artifacts on images were excluded. Three radiologists evaluated all images in consensus. Temporomandibular disc locations were classified as normal, anteriorly displaced with reduction (ADr), and anteriorly displaced without reduction (ADwr) on sagittal oblique T1-weighted images. Condylar shapes were classified as flat, rounded, and angled, and condyle anteroposterior width (c-APW) was measured on these images in closed-mouth position.

**Results:**

Ninety six discs were in normal position (40%), 70 discs were ADr (29%), and 74 discs were ADwr (31%). Eighty-four condyles were flat (35%), 100 condyles were rounded (42%), and 56 condyles were angled (23%). Mean c-APW was 7 mm in normal joints, 5.9 mm in ADr, and 5.8 mm in ADwr joints, and it was smaller in joints with anterior disc displacement (p < 0.001). In normal joints, flat and rounded type condylar shape was more common and almost equally prevalent (44% and 43%); however, rounded type was more common among ADr (%47) and angled type was more common among ADwr joints (36%) (p = 0.008). Patients with anterior disc displacement were significantly younger from normal cases and anterior disc displacement was more common among female sex.

**Conclusion:**

Mandibular condyle shape alterations and condyle size on sagittal oblique MRI plane are associated with anterior disc displacement. Angled shape was more common among ADwr joints. Joints with anterior disc displacement had smaller c-APW than normal joints.

## 1. Introduction

Temporomandibular joint (TMJ) is a complex anatomical structure composed of an articular disc and articular surfaces made up of the mandibular condyle inferiorly, glenoid fossa and the articular eminence of the temporal bone superiorly. Abnormal interaction between any of these components results in internal derangement which is known as the most frequent cause of TMJ dysfunction and a common condition that affects nearly 20%–30% of the population [1, 2]. Temporomandibular disc displacement is the most common cause of internal derangement [3] and a great majority of the discs show anterior displacement according to the relationship of the displaced disc with the mandibular condyle [4, 5]. Condylar anatomical variants like bifid condyles and pathological conditions like aplasia/hypoplasia/hyperplasia are known to be related with TMJ dysfunction [1, 3]. Whether normal morphological features of the mandibular condyle which differs greatly between individuals and even two sides in the same individual have been related with TMJ dysfunction is still not certain and to date, and many studies using various imaging modalities and methodologies with variable results have been performed in the literature to enlighten this [6–23]. Of imaging modalities, magnetic resonance imaging (MRI) is accepted as the modality of choice for evaluating TMJ dysfunction because of its high resolution and tissue contrast. It provides anatomical information and functional evaluation through closed and open-mouth images [1, 24, 25]. Closed- and open-mouth images are usually obtained in coronal or sagittal oblique plane to correct the angulation of the condylar head. Sagittal oblique images have been regarded as the most appropriate imaging plane for the evaluation of TMJ dysfunction [23, 26]. However, in many imaging-based studies evaluating the condylar morphology, axial or coronal planes were used [11, 12, 14, 15, 17, 19]. These studies had conflicting results with regard to the relationship between condyle shapes and disc displacement [11, 15]. To our knowledge, there is no study using sagittal oblique MRI plane to evaluate condylar morphology and assessing its relationship with anterior temporomandibular disc displacement. Therefore, in this study, we aimed to evaluate whether condyle morphology on sagittal oblique plane was associated with anterior disc displacement.

## 2. Materials and methods

This retrospective study was approved by the institutional review board (protocol number: 2018/204) and followed the tenets of the Declaration of Helsinki. Requirement for informed consent from patients was waived.

### 2.1. Study population

One hundred and seventy patients who underwent temporomandibular joint MRI examination with presumed diagnosis of TMJ dysfunction in a 2-year period were retrospectively evaluated. Exclusion criteria were less than 18 years of age, severe osteoarthritic changes that would alter the normal shape of the condyle, posterior disc dislocation, tumor, abscess, history of a rheumatic disease, history of facial bone fracture, and motion artifacts on MRI that would hamper evaluation and a total of 50 patients were excluded. Overall, 120 patients were eligible for analysis.

### 2.2. MRI examination and analysis

MRI examinations were performed by using two different 1.5 Tesla systems (Siemens, Erlangen, Germany and Philips Achieva, Netherlands) by using neurovascular coil. Patients were positioned in the supine position. After a T1-weighted localizer image, coronal T1-weighted images in closed mouth position, sagittal oblique T1, T2-weighted, and proton density images in closed- and open-mouth positions were obtained. Sagittal oblique images which are used to correct for the condylar angulation on true sagittal images were planned perpendicular to the long axis of the mandibular condyle. For open-mouth positions, a biting plate was used. Imaging parameters are given in detail in Table 1.

**Figure 1:**
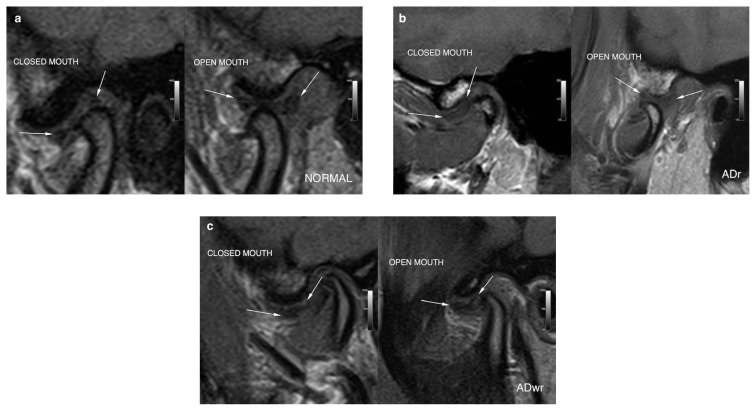
Classification of temporomandibular disc locations as normal (a), ADr (b), and ADwr (c) on sagittal oblique T1-weighted MR images.

**Figure 2:**
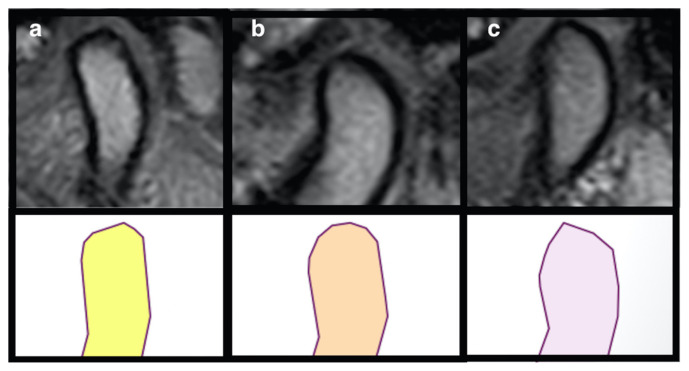
Classification of condylar shape as flat (a), rounded (b), and angled (c) on sagittal oblique T1-weighted MR images with their illustrations.

Three radiologists with different levels of experience in head and neck imaging (ME 10 years, İÇ 3 years, ATS 3 years) evaluated all images in consensus being blinded to the clinical information. Temporomandibular disc locations were classified as normal, anteriorly displaced with reduction (ADr), and anteriorly displaced without reduction (ADwr) on sagittal oblique T1-weighted image in closed-mouth position [27]. The disc position was considered normal when the intermediate zone was interposed between the condyle and the posterior slope of the articular eminence (Figure 1a). It was considered ADr when the disc was in an anterior position in relation to the condylar head and regained its normal position in open-mouth position (Figure 1b) and ADwr when the disc did not regain its normal position in open-mouth position (Figure 1c). Condylar head shapes were classified as flat, rounded, and angled on sagittal oblique T1-weighted images on the most central single slice and condyle anteroposterior width (c-APW) was also measured on these images in closed-mouth position. Condylar head was considered flat when it had a cornered appearance (Figure 2a). It was considered rounded when the superior surface had a convex appearance (Figure 2b) and angled when it had a pointy appearance with an angulation of less than 90° (Figure 2c). APW was measured at the intersection of the upper and lower half of the condyle perpendicular to the long axis of the condyle and including the bone cortices.

### 2.3. Statistical analyses

Statistical analyses were carried out using IBM SPSS V22.0. The Kolmogorov–Smirnov test was used to assess normality distribution and Levene’s test was used to assess equality of variances. Data with normal distribution were presented as means ± standard deviations and nonnormal distribution as median (interquartile range). One way ANOVA with post hoc Tukey HSD test was used to compare c-APW between three different groups. The Mann–Whitney U test was performed for comparison of age between normal cases and cases with anterior disc displacement. Pearson’s chi-squared test was used for comparison of the categorical data. Pairwise comparisons were performed with Z test with Bonferroni correction. The level of statistical significance was set as p < 0.05.

## 3. Results

In 120 patients, 240 discs and condyles were evaluated. Nineteen patients were male (16%) and 101 patients were female (84%). Median patient age was 32 years (24). Mean c-APW was 6.3 ± 1.5 mm (range: 2.9–11 mm). Of 240 discs, 96 were in normal position, 70 were ADr, and 74 were ADwr. Eighty-four condyles were flat, 100 condyles were rounded, and 56 condyles were angled. Mean c-APW was 7 ± 1.4 mm in normal cases, 5.9 ± 1.5 mm in ADr and 5.8 ± 1.4 mm in ADwr patients. In normal cases, flat and rounded type condylar shape were more common and almost equally prevalent (43% and 44%, respectively); however, rounded type was more common among ADr (%47) and angled type was more common among ADwr patients (36%) (p = 0.008) (Table 2). C-APW were significantly smaller than normal cases in patients with anterior disc displacement (p < 0.001); however, no significant condyle size difference was found between joints with ADr and ADwr (p = 0.894) (Table 3). Patients with anterior disc displacement were significantly younger than normal patients [30 (16) versus 42 (23) years, p < 0.001]. Anterior disc displacement was more common among female sex although not statistically significantly (p = 0.286) (Table 4).

## 4. Discussion

In this study, we showed that condylar morphology on sagittal oblique plane was associated with anterior disc displacement. The form and function relationship have been present in any anatomical part of the body and it is also no exception for TMJ structures. The preliminary condylar head shape classification was performed by Yale et al. as convex, flattened, angled, and rounded on coronal plane [17]. However, many following studies used different classifications with different condyle shape combinations. Some authors categorized the condyle shapes into three as convex, angled, and flat [19] or rounded, flat, and concave on coronal MRI sections [14]. We also categorized the condyles into three groups as rounded, angled, and flat on sagittal oblique MRI sections. Separate categorization of convex and rounded type by other authors was not always straightforward in our cases although we classified the condyles in consensus; therefore, we decided to unite these categories into one as rounded type. Increased prevalence of rounded condyles in our study was similar to some other studies in the literature. However, these studies used coronal MRI views to classify mandibular condyles [14, 19]. The other studies regarding condylar morphology had discrepant results but this could be explained by variable classification and methodologies [11, 12, 15, 17].Rounded condyle shape was more prevalent among ADr joints (47%) and angled type was more prevalent among ADwr joints in our study (36%). Another recent study had similar results but they evaluated condylar morphology on coronal MRI sections [11]. Sülün et al. [12], who evaluated condylar morphology on axial MRI view, found that flat condyles were associated with ADr, whereas angled condyles were associated with ADwr. Farias et al. [15] found no association with disc displacement and condylar morphology on axial and coronal MRI views. Although these studies had conflicting results with regard to the condyle morphology and ADr, their common point was that angled type condyle shape had an association with ADwr. We think that sagittal oblique plane is the most appropriate plane to evaluate the relationship between condylar shape and anterior disc displacement because physiologic gliding motion of the temporomandibular disc and disc position in relation to the condyle is only appreciated on sagittal oblique images; therefore, it makes sense that angulation of the tip of the condyle could be an additional factor to hinder the gliding and regaining its normal position.

As to the condyle size, there are also discrepancies due to interindividual variations and different methodologies. Condyle mediolateral size was 15–20 mm, and anteroposterior size was 8–10 mm according to Gray’s anatomy from axial view [28]. Yang et al. [23] measured mediolateral size as 20–21 mm and anteroposterior diameter as 9–10 mm on axial MRI views. In another CT-based study, it was 16–18 mm mediolaterally and 7–8 mm anteroposteriorly on axial sections [20]. Torres et al. [21] and Vieira-Oueiroz et al. [22] measured mediolateral diameter on coronal slices as 18–19 mm and anteroposterior diameter on axial MRI images as 5.15 and 6–7 mm, respectively. Condyle anteroposterior width on sagittal oblique MRI in our study was 6.3 mm, which is similar to the results of Vieira-Oueiroz et al. In our study, we found that patients with anterior disc displacement had smaller c-APW compared with normal patients. However, joints with ADr and ADwr did not have a significant size difference. Our finding was in line with the previous studies which also found narrower condyles in temporomandibular disc displacement [21, 22]. Ahn et al. [13] found a significant association between disc displacement and decreased total condylar volume. In their study, joints with disc displacement without reduction had the smallest condyle volumes. They explained this with condylar resorption which is characterized by progressive and repetitive bony erosion and remodeling.The marked preponderance of TMJ dysfunction in female patients is well-known [29, 30] and it was also noteworthy in our study group too, although not statistically significant. Although the exact reason for this is unknown, hormonal changes and connective tissue metabolism differences have been blamed [15, 30]. Patients with anterior disc displacement were also significantly younger than normal patients. This was also in line with the literature as internal derangement typically affects people at 20–40 years of age [1].

Our study had some limitations. Firstly, all MRI examinations were performed by a neurovascular coil rather than a TMJ-specific coil. Secondly, all MRI analyses including condyle shape classification and c-APW measurement were performed only once at a single session and interrater agreement was not performed. However, the analyses were performed in consensus by three radiologists where the final decision was given by the most experienced examiner.

In conclusion, our findings suggest that mandibular condyle shape alterations in sagittal oblique MRI plane and condyle size are significantly associated with anterior tempomandibular disc displacement. Rounded condyle shape was more common among normal and ADr joints. Angled shape was more common among ADwr joints. Joints with anterior disc displacement had smaller c-APW than normal joints.

## Figures and Tables

**Table 1:** Imaging parameters of temporomandibular MRI examinations.

Sequence	Plane	SIEMENS (1.5T)				PHILIPS (1.5T)			
		TR/TE (ms)	Slice thickness (mm)	Field of View (cm)	Matrix	TR/TE (ms)	Slice thickness (mm)	Field of View (cm)	Matrix
T2 TSE	Sagittal oblique	80/2500	3	14	256 × 256	69/3000	3	15 × 15	252 × 195
T1 TSE	Coronal	81/450	3	14	240 × 320	11/162	3	13 × 15	208 × 192
T1 TSE	Sagittal oblique	81/450	3	14	256 × 320	131/770	3	15 × 15	256 × 205
PD TSE	Sagittal oblique	21/2000	3	14	256 × 320	24/3220	3	15 × 15	184 × 147

**Table 2:** Condyle shape and prevalence in relation to temporomandibular disc positions.

Disc location	Condyle shape [number (%)]			p-value
	Rounded	Flat	Angled	
Normal	42 (44)^a,b^	41 (43)^a^	13 (13)^b^	0.008
ADr	33 (47)^a^	21 (30)^a^	16 (23)^a^	
ADwr	25 (34)^a^	22 (30)^a^	27 (36)^b^	

Columns with similar subscript letters do not differ significantly from each other at the 0.05 level.

**Table 3:** C-APW in relation to disc positions.

Condyle shape	C-APW (mm)	p-value
Normal^a^	7 ± 1.4	p < 0.001 p = 0.894
ADr^b^	5.9 ± 1.5	
ADwr^b^	5.8 ± 1.4	

Columns with similar subscript letters do not differ significantly from each other at the 0.05 level.

**Table 4:** Sex distribution and age in relation to diagnoses.

Disc location	Sex [number (%)]^a^		Age^b^ Median (IQR)	p-value
	Male	Female		p = 0.28^a^ p < 0.001^b^
Normal	8 (42)	30 (30)	42 years (23)	
Anteriorly displaced	11(58)	71 (86)	30 years (16)	

IQR: Interquartile range,

a Pearson’s chi-squared test,

b The Mann–Whitney U test
